# Melanosis coli in the elderly: a look beyond what can be seen

**DOI:** 10.1186/1471-2482-13-S1-A36

**Published:** 2013-09-16

**Authors:** Gianluca Pellino, Guido Sciaudone, Giuseppe Candilio, Antonio Camerlingo, Rosa Marcellinaro, Federica Rocco, Serena De Fatico, Silvestro Canonico, Francesco Selvaggi

**Affiliations:** 1 Unit of General and Geriatric Surgery, Second University of Naples, Naples, Italy

## Introduction

*Melanosis coli* (MC) is a benign condition found in 1% of general population. It is associated with high intake of anthranoid laxatives [1].

We herein report two cases of elderly patients with MC in whom this condition masked underlying diseases.

## Case series

### Case 1

**Figure 1:**
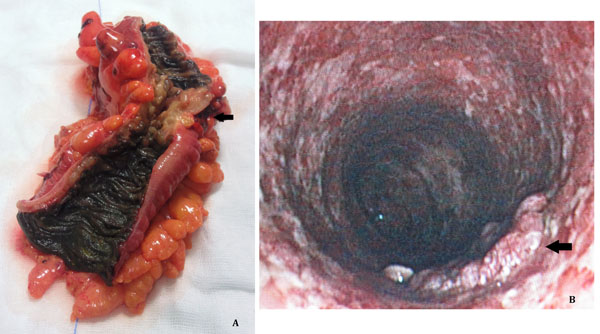
**A.** The pathological specimen of Case 1 patient. A stenosing adenocarcinoma was found at the sigmoid colon (black arrow). *Melanosis coli* was observed both in cranial and caudal segments of the colonic mucosa. **B.** Endoscopic appearance of the sigmoid colon of Case 2 patient. A severe mucosal inflammation was observed, without *Melanosis coli*. A suspect lesion was found (black arrow). Biopsies of the lesion documented a colonic adenocarcinoma.

G.T., a 75-year-old nun, came to our observation because of an history of constipation. She had been diagnosed with a constipating-like irritable bowel syndrome, but constipation had worsened in the last months and was refractory to conventional laxatives. She was taking -.. which she was self-administrating since 3 years. She had undergone a sigmoidoscopy approximately one year before, and MC was observed. At clinical exam her abdomen was distended, and she had pain at palpation in right iliac fossa. Endoscopy was advocated and a stenosis was found at the recto-sigmoid junction. MC was observed. The endoscope could not be pushed further. Biopsies were taken, and these concluded for adenocarcinoma of the colon. The patient underwent ^18^FDG-PET-CT examination, which revealed an advanced disease with secondary lesions to liver and peritoneum. The patient refused endoscopic bowel stenting and an Hartmann procedure was performed. The surgical specimen showed MC before and after the colonic stenosis (Figure 1). She received adjuvant chemotherapy. She died 48 months after surgery.

### Case 2

M. D. F., a 70-year-old woman came to our observation with an history of irritable bowel syndrome, diagnosed by General Practitioner (GP). She was used to take laxatives in case of constipation. During the last 2 years she had several episodes of diarrhoea, sometimes with emission of blood, lasting for approximately a week. Her GP prescribed suspension of laxatives and rectal enemas of Mesalazine. She underwent sigmoidoscopy, and MC was found. When we visited the lady she was having hematic liquid stool emission since one week, she was pale and weak. Inflammatory indices were high. We performed a pancolonoscopy: MC was not observed; however, an intense mucosal inflammation suggesting an inflammatory bowel disease was found extending cranially from the sigmoid colon up to the proximal transverse colon. The colon was rigid, and a lesion suspect for dysplasia-associated lesion or mass (DALM) was found in the sigmoid colon (Fig. 2). Lesion and random bioptic samples were taken. Stool cultures were negative. In the light of clinical, endoscopic and pathological findings, diagnosis of ulcerative colitis was posed. The biopsies of the lesion concluded for a adenocarcinoma. A ^18^FDG-PET-CT showed a diffuse inflammatory pattern of glucose captation of the colon. No additional features suggesting primary or secondary lesions were observed. She underwent restorative proctocolectomy with ileo-pouch-anal anastomosis and a loop-ileostomy was fashioned. The pathological exam of the entire specimen confirmed the likeliness of ulcerative colitis and the presence of the adenocarcinoma of the sigmoid colon (pT_2_N_0_). The lady underwent ileostomy closure 2 months later and she is being followed-up. No adverse events have been recorded up to 1 year after ileostomy closure.

## Discussion

Agreement exists about the benignity of MC. This condition is due to a mechanical effect of anthraquinones pigment deposition on colonic mucosa, and physiological appearance is promptly restored after laxative withdrawal within some weeks [1]. Even if authors suggested MC could harbour adenomas, evidences on such eventuality are lacking in the literature [2].

However, we are reporting these two cases to highlight the importance not to overlook mucosal alterations which can underlie MC.

In the patient described in case 1 the adenocarcinoma was likely to have been present at the time of first endoscopy: random biopsies or repetition of the procedure after laxative withdrawal could have allowed for diagnosis of the cancer at an earlier stage, with better outcomes.

Patient described in case 2 also was poorly studied before laxatives and anti-inflammatory enemas administration. As expected, MC had promptly disappeared after laxative suspension. The Mesalazine enemas conferred a falsely sane aspect to the mucosa of the distal part of the colon, but when examining the proximal part of the large bowel a severe mucosal inflammation was found. Also, an neoplastic lesion was found. Even if the diagnostic delay did not impact on surgical completeness and functional outcomes, an earlier diagnosis was desirable and achievable in this patient.

MC can be regarded as a benign condition, but one should aim to look at the underlying mucosal aspect when MC occurs. Anthranoid laxatives should be proscribed and patients encouraged to withdraw intake of these drugs.

## Competing interests

Authors have no competing interests to disclose.

## Authors’ contributions

GP designed the study, and wrote the draft of the manuscript. GS, SDF and GC collected data and participated in the drafting. SDF, AC, RM and FR collected and analyzed data. SC and FS conceived of the study, participated in its design and coordination and helped to draft the manuscript. FS performed surgical procedure in our Unit as operating surgeon. All authors read and approved the final manuscript.
